# Confidence-Guided Frame Skipping to Enhance Object Tracking Speed

**DOI:** 10.3390/s24248120

**Published:** 2024-12-19

**Authors:** Yun Gu Lee

**Affiliations:** School of Software, Kwangwoon University, Kwangwoon-ro 20, Nowon-gu, Seoul 01897, Republic of Korea; harmony96@gmail.com; Tel.: +82-2-940-8112

**Keywords:** visual tracking, object tracking, fast object tracking, online tracking

## Abstract

Object tracking is a challenging task in computer vision. While simple tracking methods offer fast speeds, they often fail to track targets. To address this issue, traditional methods typically rely on complex algorithms. This study presents a novel approach to enhance object tracking speed via confidence-guided frame skipping. The proposed method is strategically designed to complement existing methods. Initially, lightweight tracking is used to track a target. Only in scenarios where it fails to track is an existing, robust but complex algorithm used. The contribution of this study lies in the proposed confidence assessment of the lightweight tracking’s results. The proposed method determines the need for intervention by the robust algorithm based on the predicted confidence level. This two-tiered approach significantly enhances tracking speed by leveraging the lightweight method for straightforward situations and the robust algorithm for challenging scenarios. Experimental results demonstrate the effectiveness of the proposed approach in enhancing tracking speed.

## 1. Introduction

Object tracking is an important task in computer vision with diverse applications, including autonomous vehicle driving [1], surveillance [2], sports video analysis [3], and human–computer interaction [4]. Despite significant recent advancements, object tracking remains challenging owing to various obstacles, including illumination variation, occlusion, background clutter, target deformation, similar objects, scale transformation, low resolution, and fast motion [5].

In single-object tracking, the initial target is provided in the first frame, and the method’s objective is to locate the specific target and trace its trajectory as it moves through a sequence of frames within a video. Traditional methods for object tracking often relied on hand-crafted features, such as the histogram of oriented gradients (HoG) [6], to estimate the target’s position across frames. However, these approaches may struggle to interpret semantic target information and effectively handle significant changes in appearance [7]. Recently, deep learning-based methods have gained increasing attention for more robust and accurate tracking solutions in the field of object tracking. Numerous object tracking architectures have been developed based on convolutional neural networks (CNNs) [8,9,10,11,12,13,14,15], siamese neural networks (SNNs) [16,17,18,19], recurrent neural networks (RNNs) [20], generative adversarial networks (GANs) [21], and MixFormer [22,23].

Given that real-time object tracking is crucial for several practical applications, numerous object tracking methods have been proposed [24,25,26,27]. Although these methods enable real-time tracking, their processing speeds must be improved. This is particularly crucial when various computer vision algorithms, including object tracking, coexist and run on hardware with limited computing resources. Efficient processing is necessary to prevent object tracking methods from monopolizing the available computing resources. Additionally, some algorithms are restricted to real-time execution on expensive high-end GPUs. Consequently, achieving higher processing speeds in object tracking is imperative as it contributes to the seamless and concurrent operation of diverse algorithms in resource-constrained environments.

Object tracking is challenging owing to factors such as occlusion, illumination variation, and target deformation as mentioned previously. To tackle these issues, researchers have developed robust algorithms that are inevitably complex. Moreover, to maintain high tracking accuracy in real-time object tracking, these challenging scenarios must be considered. Hence, real-time tracking methods also tend to be complex and resource-intensive. However, not every frame features target objects embroiled in such intricate circumstances. Several instances involve only minor changes in the target’s behavior between consecutive frames. When the camera motion remains minimal, targets within successive frames remain relatively stationary. Thus, applying a complex, resource-heavy algorithm to such straightforward scenarios results in the inefficient use of computing resources. A more efficient approach involves selectively deploying a straightforward algorithm for such cases, essentially reserving the use of complex algorithms exclusively for frames encompassing difficult situations. This approach can effectively enhance the tracking speed while preserving accuracy. The key challenge in this approach lies in discriminating between easy and difficult situations with minimal computational cost.

Therefore, this study introduces a novel approach aimed at accelerating the object tracking speed by selectively applying complex object tracking to only frames containing difficult targets. The proposed method is not intended for a standalone operation; rather, it is designed to complement existing methods synergistically. The proposed method initially attempts to track a target using a lightweight object tracking method with an extremely small computational load, which is based on the block-matching algorithm [28]. Subsequently, the proposed method evaluates the tracking results using a newly introduced confidence level. For cases where the tracking results of the lightweight tracking are deemed unsuccessful, a robust algorithm (an existing technique) intervenes to track the target. The proposed method is designed to be easily integrated with existing methods, and this study provides an integration example in detail. This two-tiered approach effectively enhances tracking speed.

The remainder of this paper is structured as follows: Section 2 provides a concise summary of related work. Section 3 discusses the proposed algorithm in detail. Section 3.3 discusses the integration of the proposed method with an existing technique. Section 4 presents the experimental results. Finally, Section 5 presents the conclusions of the study.

## 2. Related Works

The block-matching algorithm [28] stands as a fundamental tool extensively employed for estimating motion between successive frames of video sequences. This technique finds applications in object tracking, as demonstrated in [29,30]. El-Azim et al. tracked a single moving object within a frame under the assumption that an object is a rigid body [29]. Hariharakrishnan also introduced a fast object tracking algorithm using adaptive block matching [31]. One of the key advantages of object tracking based on the block-matching algorithm is its simplicity and computational efficiency. However, despite its high processing speed, this technique has not seen widespread adoption in recent research due to limited performance.

The advent of the deep learning revolution [32] has not only transformed object recognition [33] but has also generated considerable interest in their application in object tracking. This evolution has led to the emergence of numerous tracking methodologies based on CNNs [8,9,10,11,12,13,14,15]. By leveraging the breakthroughs in CNN architectures, these trackers capitalize on their inherent advantages. They effectively capture and encode the distinctive characteristics of objects as high-dimensional features by harnessing the potent representational capabilities of CNNs. Numerous studies [8,9,10,11,12,13,14,15] have demonstrated that these feature representations can be efficiently used for object tracking.

Although CNN-based trackers are widely employed, they have certain limitations [7]. To address these limitations, recent studies have focused on Siamese neural networks [16,17,18,19]. Siamese-based trackers conceptualize object tracking as the learning of a similarity map between the target template and candidate search regions in subsequent frames [34] while harnessing the advantages of deep networks for end-to-end learning. Siamese neural network-based trackers have garnered considerable attention because of their balanced accuracy and computational effectiveness [35]. Thus, they are considered among the most promising architectures for object tracking [36]. The Siamese Region Proposal Network (SiamRPN) [17] employs the concept of the region proposal network from [37]. Li [34] introduced the Siamese Region Proposal Network++ (SiamRPN++), which employs ResNet [38] as its backbone network.

## 3. Proposed Algorithm

**Figure 1:**
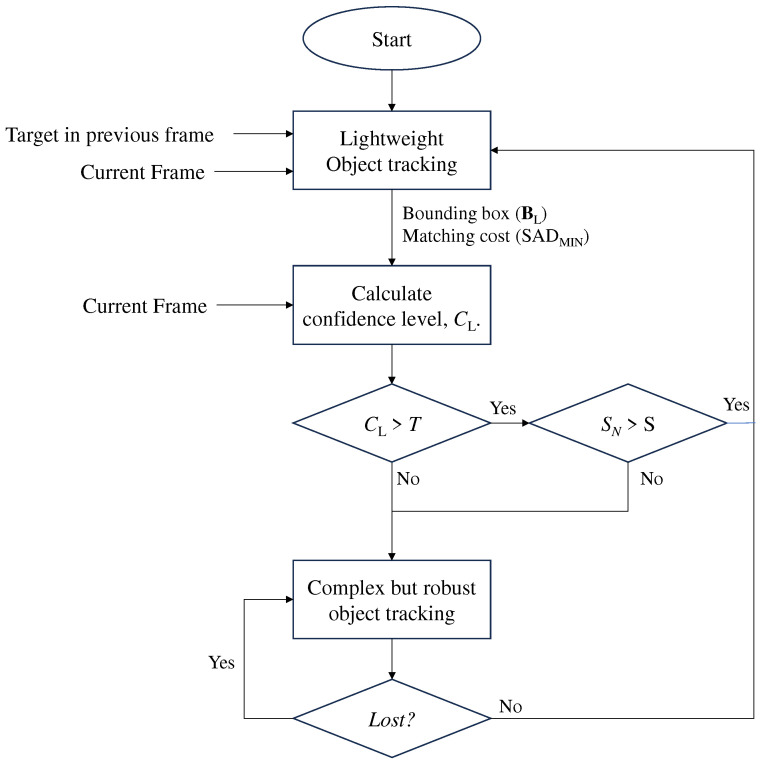
Overview of the proposed method. *T* is a threshold value. $S_{N}$ denotes the maximum number of consecutive frames processed without invoking complex but robust object tracking, while *S* indicates the number of consecutive frames processed following the activation of complex but robust object tracking.

Figure 1 provides an overview of the proposed algorithm. Initially, the lightweight object tracking method, with the block-matching algorithm [28], is used to track a target within the current frame, outputting a bounding box of the target ($B_{L}$), along with its matching cost (${\mathrm{SAD}}_{\mathrm{MIN}}$). Although the computational complexity of lightweight object tracking is minimal, the predicted $B_{L}$ is not highly reliable. To address this limitation, we calculated the proposed confidence level associated with the predicted bounding box ($B_{L}$) using the pixels of the target in the previous frame and the matching cost obtained from lightweight object tracking. Subsequently, the proposed algorithm assesses whether the confidence level surpasses a specified threshold. If it does, the bounding box predicted by the lightweight object tracking method is considered the final output. Otherwise, the proposed algorithm invokes an existing method that ensures reliable results in challenging scenarios. When the tracked object disappears, the confidence level typically drops due to inconsistencies in the tracking results from the lightweight object tracking. This will trigger the robust tracker to reinitialize tracking when the object reappears. If lightweight tracking mistakenly locks onto another object, the confidence evaluation mechanism detects the mismatch based on predefined thresholds, prompting a corrective action by the robust tracker. It should also be noted that when the robust tracker fails to track the target due to object disappearance, the next frame is tracked using the robust tracker again. Additionally, after the lightweight object tracker processes $S_{N}$ consecutive frames, the robust tracker is forcibly invoked to ensure long-term reliability. Here, $S_{N}$ denotes the maximum number of consecutive frames processed without invoking complex but robust object tracking, and *S* represents the number of consecutive frames processed following the activation of complex but robust object tracking.

Two essential factors must be considered for achieving this. First, lightweight object tracking should be significantly faster than existing methods to ensure that its computational cost can be neglected. If the complexity of lightweight object tracking becomes comparable to that of existing methods, the proposed approach cannot effectively accelerate the object tracking speed. The second factor is the reliability of the calculated confidence level. Ensuring a reliable confidence calculation is crucial because high confidence in incorrect tracking results of lightweight object tracking can lead to significantly poor accuracy in the final object tracking results.

### 3.1. Lightweight Object Tracking

Not all target objects present challenging situations. In several cases, the changes in a target between successive frames are relatively small. Moreover, when the camera is static and the target is motionless, the target in successive frames also remains static. Hence, lightweight object tracking focuses on accurate target tracking in simpler scenarios with fewer changes between successive frames. One effective method for achieving this is using a block-matching algorithm [28]. This work employs a simple block-matching apporach without considering advanced techniques such as the adaptive block-matching method described in [31].

**Figure 2:**
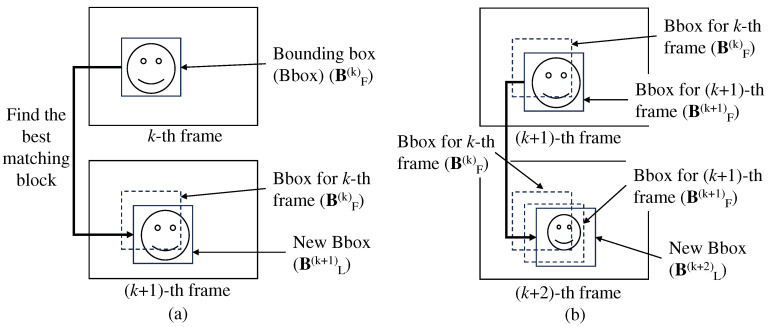
Lightweight object tracker using a block-matching algorithm: (**a**) The method successfully finds the target in the (*k* + 1)-th frame. (**b**) If $B_{L}^{\left(k+1\right)}$ is determined as a final bounding box for the (*k* + 1)-th frame ($B_{F}^{\left(k+1\right)}=B_{L}^{\left(k+1\right)}$), the method finds the target in the (*k* + 2)-th frame using the target predicted in the (*k* + 1)-th frame.

Figure 2a illustrates the lightweight object tracking method, which employs a block-matching algorithm. The best match for the bounding box in the *k*-th frame, $B_{F}^{\left(k\right)}$, is found in a search area in the (*k* + 1)-th frame, and this position is set as the position of the new bounding box, $B_{L}^{\left(k+1\right)}$ in the (*k* + 1)-th frame. The size of the new bounding box $B_{L}^{\left(k+1\right)}$ remains unchanged from $B_{F}^{\left(k\right)}$ in the *k*-th frame.

(1) $$\left(d_{x},d_{y}\right)=\arg\min_{(m,n)\in \mathrm{SR}}\mathrm{SAD}\left(m,n\right)$$

(2) $$SAD\left(m,n\right)=\sum_{j=b_{y}}^{b_{y}+B_{H}}\sum_{i=b_{x}}^{b_{x}+B_{W}}|I^{k}\left(i,j\right)-I^{k+1}\left(i+m,j+n\right)|$$

Here, $I^{k}\left(i,j\right)$ is the pixel value at (i,j) in the *k*-th frame. $\left(d_{x},d_{y}\right)$ is the displacement of the target from the *k*-th frame to (*k* + 1)-th frame, and SR is the search range. Equation (1) represents the full search method, which examines all the positions in the search area. However, fast block-matching algorithms, which examine a limited set of search points, can be considered to reduce the computational burden of lightweight object tracking. These fast algorithms are discussed in the Experimental Results section. ($b_{x}$, $b_{y}$) is the coordinate of the bounding box at the upper left corner. $B_{W}$ and $B_{H}$ are the width and height of the bounding box, respectively. If $\left(b_{x},b_{y},B_{W},B_{H}\right)$ is a bounding box for $B_{F}^{\left(k\right)}$, the bounding box for the (*k* + 1)-th frame, $B_{L}^{\left(k+1\right)}$ is $\left(b_{x}+d_{x},b_{y}+d_{y},B_{W},B_{H}\right)$. The lightweight object tracking method predicts only the displacement. The value of the sum of absolute difference (SAD) at $\left(d_{x},d_{y}\right)$, ${\mathrm{SAD}}_{\mathrm{MIN}}$, is used in confidence level evaluation, as depicted in Figure 1.

RGB color space is utilized to achieve more accurate prediction results. Then, the pixel value difference in Equation (2) is defined as follows:

(3) $$|I^{k}-I^{k+1}\left|=\right|I_{R}^{k}-I_{R}^{k+1}\left|+\right|I_{G}^{k}-I_{G}^{k+1}\left|+\right|I_{B}^{k}-I_{B}^{k+1}|$$

Here, $I_{R}$, $I_{G}$, and $I_{B}$ represent the red, green, and blue pixel components, respectively. For convenience, the notation (x,y) is omitted.

As described in Section 2, object tracking based on the block-matching algorithm offers limited performance. The accuracy of its performance needs to be evaluated through an experiment. Frames with even indices (2i) were tracked using SiamRPN++ [34], whereas frames with odd indices (2i+1) were tracked using the lightweight tracking method. The tracking process is as follows: The ground-truth bounding box for the target in the first frame is given as $B_{GT}^{\left(1\right)}$. Applying SiamRPN++ to the second frame (or 2*i*-th frame) results in the bounding box for the second frame, $B_{F}^{\left(2\right)}$. The lightweight tracking finds the best matching bounding box within the third frame (or (2*i* + 1)-th frame), $B_{L}^{\left(3\right)}$. The size of the bounding box in the lightweight tracking method remains the same as that used for the second frame.

In this experiment, the VOT2018 [39] dataset, comprising 60 videos with a total of 21,356 frames, was used for short-term single-object tracking. The search range for the block-matching algorithm in the lightweight tracking method was set to ±16. Table 1 presents the performance of the lightweight tracking method integrated with SiamRPN++ (LTS). Owing to its limited capability in handling various challenging scenarios mentioned earlier, the performance of the LTS is expected to be significantly poor compared with SimaRPN++ in terms of accuracy, robustness, lost frame number, and expected average overlap (EAO). Nevertheless, the target was lost only in 95 of the 21,356 frames. These results indicate the effectiveness of lightweight tracking based on the block-matching algorithm in various cases. The subsequent subsection proposes a method to evaluate the accuracy of the tracking results of lightweight tracking. Hence, frames that fail to track the target or degrade the tracking performance can be selectively retracked using SiamRPN++. This approach can improve processing speed while preserving tracking performance.

### 3.2. Evaluation of Lightweight Object Tracking Result

The movement of a target in a video can include various challenging scenarios, as well as scenarios in which the target is either static or undergoes subtle changes in position or appearance. The lightweight object tracking is responsible for accurately and rapidly tracking targets in simpler scenarios with minimal changes between successive frames. In challenging scenarios, lightweight object tracking may encounter difficulties and fail to track the target, thus necessitating the evaluation of tracking results to ensure reliability. To evaluate the accuracy of the tracked bounding box ($B_{L}$) in the current frame, the pixel-wise similarity between $B_{L}$ in the current frame and ($B_{F}$) in the previous frame must be measured. Although numerous studies have attempted to estimate the similarity of two blocks or objects, incorporating several of these methods introduces additional complexity, which is undesirable.

Instead, a straightforward approach for examining the pixel-wise similarity involves comparing the pixel differences between the two bounding boxes, which are already calculated during lightweight object tracking. This difference, denoted as ${\mathrm{SAD}}_{\mathrm{MIN}}$, is the output of the lightweight object tracking (as shown in Figure 1) and corresponds to the value of the sum of absolute differences (SAD) from Equation (2) at $B_{L}$, i.e., $\left(d_{x},d_{y}\right)$. A small value of ${\mathrm{SAD}}_{\mathrm{MIN}}$ indicates that the bounding boxes are well matched, the tracking results from the lightweight object tracking are reliable, and $B_{L}$ can be considered the final bounding box for the current frame.

However, relying solely on the SAD value to determine accuracy may result in poor performance. For example, if the target includes complex textures, the SAD value may be large even if the matching is accurate, whereas a homogeneous target with fewer textures may have a small SAD value, even in an unmatched case. Therefore, texture must be considered when determining accuracy. The amount of texture is concurrently considered by analyzing the gradients of the bounding boxes. This study proposes a method to evaluate the accuracy of $B_{L}$ using a confidence level ($C_{L}$) as follows:

(4) $$C_{L}=\min\left(\frac{G_{X}}{{\mathrm{SAD}}_{\mathrm{MIN}}},\frac{G_{Y}}{{\mathrm{SAD}}_{\mathrm{MIN}}}\right)$$

Here, $G_{X}$ and $G_{Y}$ are gradient values calculated using pixels in $B_{F}$ as follows:

(5) $$G_{X}=\sum_{\left(i,j\right)\in B_{F}}\left|I\left(i+1,j\right)-I\left(i,j\right)\right|$$

(6) $$G_{Y}=\sum_{\left(i,j\right)\in B_{F}}\left|I\left(1,j+1\right)-I\left(i,j\right)\right|$$

As the lightweight object tracking calculates SAD in RGB color space, as shown in Equations (1) and (3), the two aforementioned gradient values, $G_{X}$ and $G_{Y}$, are also calculated in RGB color space.

Objects with complex textures have a larger gradient value, whereas objects with simple textures have a smaller gradient value. Hence, for the same SAD value, the confidence level increases as the gradient values increase. Herein, this reliability was assessed separately along the x and y directions, and the confidence level was determined as the minimum of these two values. Finally, as shown in Figure 1, if the confidence level exceeds the threshold value, the bounding box, $B_{L}$ is considered final. Otherwise, the tracking result of the lightweight object tracking is considered unsuccessful, and a more accurate and complex method is employed for target tracking. The performance in terms of accuracy and speed gain, depending on the threshold value is provided in the Experimental Results section.

Let $B_{F}^{\left(k\right)}$ and $B_{L}^{\left(k+1\right)}$ be the final bounding box for the *k*-th frame and the bounding box predicted from lightweight object tracking, respectively. If $B_{L}^{\left(k+1\right)}$ is determined as the final bounding box according to the confidence level, then $B_{F}^{\left(k+1\right)}$ is set to $B_{L}^{\left(k+1\right)}$. The block-matching algorithm used in the lightweight object tracking method finds the best matching bounding box of $B_{F}^{\left(k+1\right)}$ in the (*k* + 2)-th frame as depicted in Figure 2b.

During the evaluation of lightweight object tracking results, two gradients, $G_{X}$ and $G_{Y}$, must be calculated. However, the computational cost for calculating these gradients is approximately equal to only two-point checks in the block-matching algorithm used in lightweight object tracking. Considering that the number of points to be examined in lightweight object tracking is significantly larger than just two points, the computational burden of calculating the two gradient values can be considered negligible compared to the overall computational complexity of lightweight object tracking.

### 3.3. Integration

This section explains the integration of the proposed method with SiamRPN++ [34] where SiamRPN++ is one of the state-of-the-art methods for object tracking algorithms.

**Figure 3:**
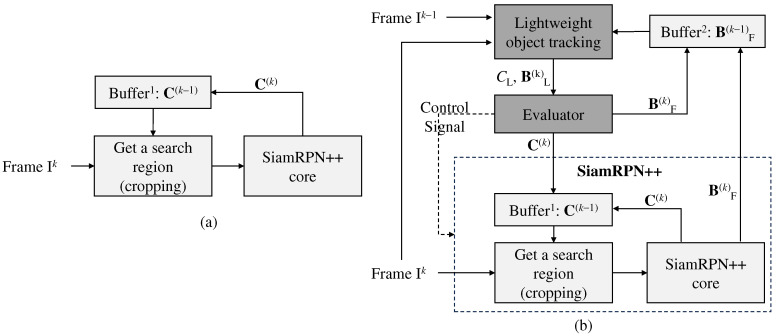
(**a**) Before applying the proposed algorithm to SiamRPN++ and (**b**) after applying the proposed algorithm to SiamRPN++. $C^{k}$ is the center position of the bounding box $B_{F}^{\left(k\right)}$.

Figure 3a,b illustrate the difference before and after applying the proposed method to SiamRPN++. The figures depict only those components that are involved in integration with the proposed method. In Figure 3a, SiamRPN++ crops a search region from the *k*-th frame, $I^{k}$, according to $C^{\left(k-1\right)}$ (the center position of the bounding box $B_{F}^{\left(k-1\right)}$). As the SiamRPN++ core tracks the target within the cropped search region, the new center position, denoted as $C^{\left(k\right)}$, is updated in ${\mathrm{buffer}}^{1}$. The proposed method can be integrated with SiamRPN++, as shown in Figure 3b. The lightweight object tracking tracks the target using $I^{k-1}$, $I^{k}$, and $B_{F}^{\left(k-1\right)}$ and outputs the confidence level, $C_{L}$, and the predicted bounding box $B_{L}^{\left(k\right)}$. Based on the confidence level, the evaluator determines the success of tracking. When the tracking is successful, $B_{L}^{\left(k\right)}$ is considered the final bounding box, $B_{F}^{\left(k\right)}$, and the method updates ${\mathrm{buffer}}^{2}$ with the final bounding box. Furthermore, the evaluator should update ${\mathrm{buffer}}^{1}$ with $C^{\left(k\right)}$, which represents the center position of $B_{F}^{\left(k\right)}$. As SiamRPN++ crops the search region according to the center of the tracked bounding box, ${\mathrm{buffer}}^{1}$ maintains the center position of the bounding box for the most recently tracked target. If the lightweight object tracking method proves to be ineffective, SiamRPN++ is activated to accurately track the target. In this case, the bounding box from SiamRPN++ is updated to ${\mathrm{buffer}}^{2}$ as the lightweight object tracking requires the bounding box for tracking the next frame.

The parameter $S_{N}$ suggests the maximum number of consecutive frames for which lightweight object tracking is employed without performing SiamRPN++. If the tracking results from the lightweight object tracking are used as the final bounding boxes for $S_{N}$ consecutive frames, then the next frame is forcibly tracked using SiamRPN++.

## 4. Experimental Results

The proposed method was developed to enhance the speed of object tracking by combining it with existing algorithms, which is a novel approach in this field. Comparing the proposed method with existing techniques is challenging as it cannot be used independently and must be used with the existing algorithms. Hence, this study evaluated the performance of the proposed algorithm by comparing the tracking speed and accuracy before and after applying the proposed algorithm to existing methods. The processing speed was evaluated using a personal computer with an AMD Ryzen Threadripper 1900X (3.8 GHz), NVIDIA Geforce RTX 2080 Ti (11 GB VRAM), and 96 GB of RAM. The lightweight object tracking method was executed on the CPU, while SiamRPN++ and MixFormer [40] were executed on the GPU.

### 4.1. Implementation of Lightweight Object Tracking

The purpose of lightweight object tracking is to track the target in scenarios with minimal changes between successive frames. Hence, for lightweight object tracking, a small search area is sufficient to track the target. In the experiments, the search range for object tracking was set to ±16 to examine only a small search area. The total number of search points within the search area was ${\left(16\times 2+1\right)}^{2}=1089$.

Single-object tracking involves tracking only one object in each frame. Hence, the total number of search points for each frame is only ${\left(16\times 2+1\right)}^{2}=1089$. The computational burden of the full-search block-matching algorithm, as expressed in Equation (1), where all search points are exhaustively examined individually, is negligible in modern computing devices. Also, the computational burden of lightweight object tracking can be reduced by employing fast block-matching algorithms. To further reduce computing costs, Intel Advanced Vector Extensions 2 (Intel AVX2) can be utilized to implement the SAD function, as shown in Equation (2).

### 4.2. Proposed Method with SimaRPN++

To access object tracking, the foundational algorithm chosen was SiamRPN++. The integration of the proposed algorithm with SiamRPN++ is outlined in detail in Section 3.3. The VOT2018 [39] dataset containing 60 videos with 21,296 frames was used in the experiments for short-term single object tracking. To merge the proposed method with SiamRPN++, the foundational code for SiamRPN++ was sourced from [41]. SiamRPN++ and the proposed method were executed on GPU and CPU, respectively.

Table 2 presents a comparison of the performance metrics among different methods: $M_{\mathrm{BASE}}$ (SiamRPN++ using ResNet backbone), frame skipping ($M_{\mathrm{SKIP}}$), and the proposed method without confidence level evaluation ($M_{P1}$) and with confidence level evaluation ($M_{P2}$). $S_{N}$ denotes the maximum number of frames skipped consecutively without performing SiamRPN++. Abbreviations A, R, L, EOA, S, and FPS correspond to accuracy, robustness, lost count, expected average overlap, skip, and frames per second, respectively. For $M_{\mathrm{SKIP}}$, the initial frame was tracked using SimaRPN++. The subsequent $S_{N}$ frames were skipped without tracking, and their bounding boxes were replicated from the bounding box of the initial frame. For instance, with $S_{N}=3$, only frames indexed as 1, 5, 9, 13, etc., underwent tracking using SiamRPN++, whereas frames (2, 3, 4), (6, 7, 8), etc., were skipped. The table indicates that increasing $S_{N}$ significantly enhances the processing speed. While SiamRPN++($M_{\mathrm{BASE}}$) operated at 58.3 FPS, $M_{\mathrm{SKIP}}$ with $S_{N}=10$ achieved 428.2 FPS. However, as $S_{N}$ increased, the accuracy, robustness, and EOA metrics decreased, thus indicating that the bounding box of the previous frame is often not applicable to future frames. By contrast, $M_{P1}$ involves tracking these frames using the lightweight object tracking method, which predicts the position of the bounding box. This prediction enhances accuracy, robustness, and EOA performance over $M_{\mathrm{SKIP}}$. Although this method outperforms $M_{\mathrm{SKIP}}$, the bounding boxes predicted inaccurately can still lead to performance degradation compared with SiamRPN++.

In $M_{P2}$, SiamRPN++ is applied to frames with a confidence level below a given threshold. Hence, the value of S(%) for $M_{P2}$ was lower than that for $M_{P1}$, as presented in Table 2. For instance, with $S_{N}=1$ and T = 0.5, S(%) values for $M_{P1}$ and $M_{P2}$ were 49.8 and 34.1, respectively. This indicates that approximately 15.7% of the total frames with a confidence level below the threshold employed SiamRPN++ to accurately track the target. This process significantly improved the performance of $M_{P2}$ in terms of accuracy, robustness, and EOA, compared with $M_{P1}$. This demonstrates that the proposed confidence level was an effective threshold for the accuracy of the predicted bounding boxes. Comparing $M_{P2}$ (T = 0.5 and $S_{N}=1$) with SiamRPN++ ($M_{\mathrm{BASE}}$), the proposed $M_{P2}$ accelerated the processing speed by approximately 1.5 times, whereas their accuracies were almost identical. However, the robustness and EOA of $M_{P2}$ (T = 0.5 and $S_{N}=1$) were slightly lower than those of SiamRPN++. This slight degradation may stem from an increase in the seven lost frames in $M_{P2}$ and the absence of bounding box size updates in the lightweight object tracking method. In $M_{P1}$, as $S_{N}$ increased, the accuracy, robustness, lost counts, and EOA performance degraded significantly. However, in $M_{P2}$, even as $S_{N}$ increased, the decline in performance metrics was relatively minimal. For example, in $M_{P1}$, when $S_{N}$ was 1, the number of lost counts was 87; however, when $S_{N}$ increased to 10, the number of lost counts increased by approximately 2.6 times to 225. This indicates that indiscriminate frame skipping negatively impacted tracking performance. By contrast, for $M_{P2}$, when $S_{N}$ was 1, the number of lost counts was 59, and when $S_{N}$ increased to 10, the number of lost counts only increased to 84, approximately 1.42 times higher. This demonstrates that the confidence evaluation effectively identifies unreliable frames and triggers robust tracking to prevent tracking failures.

Table 3 lists the performance variation with respect to different threshold values. As the threshold value decreased, the S(%) value increased. An important observation is that $M_{P2}$ with $S_{N}$ = 1 and T = 0.67 outperformed SiamRPN++ ($M_{\mathrm{BASE}}$) in terms of accuracy, robustness, lost counts, and EOA by a small margin. Although SiamRPN++ yielded better tracking results than the lightweight object tracking method, there were instances in which the latter performed better. A threshold value of 0.67 with $S_{N}$ = 1 corresponded to a scenario where $M_{P2}$ matched or slightly surpassed the performance of SiamRPN++ in terms of accuracy, robustness, lost counts, and EOA. (Note that the processing speed of $M_{P2}$ is significantly higher than that of SiamRPN++.) Despite extensive experiments, determining the optimal threshold value remains a challenge. High threshold values lead to a random performance owing to the factors mentioned earlier. For instance, the threshold value of 0.33 demonstrated superior performance not only in terms of speed but also in terms of accuracy, robustness, lost counts, and EOA as compared with the threshold values of 0.4 and 0.5 (Table 3). However, as the threshold value decreased further, the performance of accuracy, robustness, lost counts, and EOA deteriorated.

Table 4 presents a comparison of $M_{P2}$ with $M_{\mathrm{BASE}}$ in terms of FPS for some sequences. For $M_{\mathrm{BASE}}$ (SiamRPN++), the difference in FPS across the sequences was not pronounced. The highest speed, 63.7 FPS, was achieved in the *ant3* sequence, whereas the lowest speed, 51.8 FPS, was observed in the *ant1* sequence. By contrast, for $M_{P2}$, the variance in FPS across the sequences was significant. The highest speed, 117 FPS, was reached in the *handball1* sequence, whereas the lowest speed, 58.9 FPS, was recorded in the *book* sequence. Here, $S_{N}$ and T were set to 1 and 0.5, respectively. The speed of the proposed method depends on the degree of change between successive frames within a sequence. When the target’s motion is relatively small, the lightweight tracking method accurately tracks the target and achieves significant speed gains. However, when the target’s motion is substantial, the proposed method results in processing overhead without corresponding improvements in speed. For example, in the *book* sequence, the target features highly complex motion. As lightweight object tracking relies on a block-matching algorithm that solely considers translational motion, accurately tracking a rotating book becomes challenging. Owing to the inaccurate tracking results, the confidence levels dropped below the threshold, and the tracking speed did not improve. By contrast, in the *fish1* sequence, the target (a fish) exhibited minimal motion. The confidence levels surpassed the threshold, and the proposed method skipped executing SiamRPN++ for most frames, thus achieving maximum acceleration.

Li [34] proposed a fast variant of SiamRPN++ using MobileNet [42] backbone, denoted as $M_{\mathrm{Mobile}}$ in Table 5. This SiamRPN++ variant ($M_{\mathrm{Mobile}}$) enhances processing speed compared with $M_{\mathrm{BASE}}$ (SiamRPN++). In $M_{P3}$, the proposed method is combined with $M_{\mathrm{Mobile}}$ to further increase the tracking speed. Table 5 lists the tracking speed enhancement of $M_{P3}$ using the proposed method at various threshold values. As presented in the table, $M_{\mathrm{Mobile}}$ improved the tracking speed over $M_{\mathrm{BASE}}$ while providing comparable performance. $M_{P3}$ further improved the tracking speed of $M_{\mathrm{Mobile}}$ while minimizing performance degradation. Notably, when $S_{N}$ = 1 and T = 0.33, $M_{P3}$ achieved performance accuracy comparable to $M_{\mathrm{Mobile}}$ while improving the tracking speed by approximately 1.64 times.

Given the compact size of the target objects in VOT2018, the 3% overhead incurred by lightweight object tracking, which employs a full-search block-matching algorithm implemented using C code, was practically negligible. However, when dealing with larger target sizes, the search range may need to be expanded. In such cases, utilizing fast search techniques is essential.

### 4.3. Proposed Method Combined with Other Methods Including MixFormer

As depicted in Figure 1, the lightweight object tracking method was used to find the best candidate for a target from the previous frame within the current frame. Subsequently, the confidence level, $C_{L}$, was calculated and used to determine whether to invoke a robust but complex existing method. Therefore, for a given target, the existing method did not influence the skipping decision. The only impact of the existing method was that if it failed to detect the target object, an incorrect target object was fed into the lightweight object tracking model. Consequently, the performance of the proposed method is not dependent on the specific existing method combined with it. Instead, the major factor affecting the performance of the proposed method is the characteristics of the input video, such as texture, motion amount, object deformation, and other factors.

To validate this, two experiments were designed as follows: In the first experiment, we assumed the existence of an ultimate tracking algorithm, $U_{BASE}$, that always provided the ground truth. The proposed method combined with $U_{BASE}$ is denoted as $U_{P}$. Given the ground truth for the dataset, $U_{BASE}$ consistently provides the correct tracking results for the corresponding frames. Since recent tracking methods generally outperform $M_{BASE}$ (SiamRPN++), their performance will likely fall between that of $M_{BASE}$ and $U_{BASE}$. In the second experiment, the proposed method was combined with the real tracking algorithm, MixFormer [22,23]; the source code for MixFormer is available in [40]. In the proposed method combined with MixFormer, $MF_{BASE}$ is denoted as $MF_{P}$. Since the optimal parameters for MixFormer on the VOT2018 dataset were not specified, default values were used in this experiment.

Table 6 shows the comparison results of $M_{P2}$, $U_{P}$, and $MF_{P}$ in terms of skipping rate (%) for $S_{N}$ = 1, 2, and 5. The threshold value was set to 0.5. The table shows the average skipping rates across the entire VOT2018 dataset and presents the skipping rates for selected video sequences from the dataset. As shown in the table, the average skipping rates for $S_{N}$ = 1, 2, and 5 were approximately 0.34, 0.45, and 0.55, respectively, regardless of the algorithms used. Despite the significantly superior tracking performance of $U_{BASE}$ compared to $M_{BASE}$ and $MF_{BASE}$, the average skipping rates across the three methods were similar. This observation underscores that the choice of algorithm has minimal impact on the skipping decision. Instead, the skipping rate is highly influenced by the characteristics of the video sequence. For example, the skipping rates for the *fish1* sequence at $S_{N}$ = 2 were 0.63, 0.65, and 0.65 for $M_{P2}$, $U_{P}$, and $MF_{P}$, respectively. On the other hand, the skipping rates for the *book* sequence for $S_{N}$ = 2 were 0.05, 0.06, and 0.03 for the same methods, respectively.

Table 7 represents the accuracy, robustness, and EOA for $M_{P2}$, $U_{P}$, and $MF_{F}$. Since $U_{BASE}$ always provided the correct tracking results, there were no lost frames, resulting in a robustness value of 0 for $U_{P}$. Although the proposed algorithm accelerated the tracking speed by frame skipping, the performance degradation remained minimal.

The ground truth represents the theoretical upper bound of tracking performance, as no tracking algorithm can outperform it. By combining the proposed method with ground truth, the experiments in Table 6 and Table 7 demonstrate the maximum potential performance when integrated with an ideal tracker. These experimental results show that the proposed method provides consistent performance regardless of the combined existing methods, both in terms of accuracy and acceleration.

To further validate the proposed method, experiments were conducted on two additional widely used datasets, namely OTB100 [5] and UAV123 [43], using MixFormer as the baseline tracker, as presented in Table 8. Compared to $MF_{BASE}$, the MixFormer combined with the proposed method, $MF_{P}$ significantly accelerates processing speed with only minimal degradation in AUC score and precision for the OTB100 dataset. Interestingly, for the UAV123 dataset, the proposed method ($MF_{P}$) not only accelerated the processing speed but also slightly improved tracking performance (AUC and precision). This improvement may be attributed to the characteristics of UAV123, where objects exhibit relatively predictable motion and minimal background changes, allowing the lightweight tracker to perform effectively even with frame skipping. These results confirm that the proposed method is not limited to specific datasets but is effective across various tracking scenarios, highlighting its generalizability and robustness.

**Figure 4:**
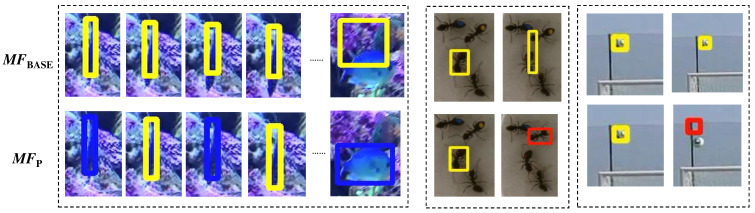
Examples of successful and unsuccessful tracking. Yellow and green rectangles represent results from MixFormer and lightweight trackers, respectively. Red rectangles show unsuccessful tracking results.

Figure 4 illustrates examples of successful and unsuccessful tracking. In the *fish* sequence, MixFormer ($MF_{BASE}$) slightly missed the target across the frames, while MixFormer combined with the proposed method ($MF_{P}$) accurately tracked the target throughout the sequence. This highlights how the lightweight tracker can enhance tracking accuracy in some scenarios. Conversely, in the *ant1* sequence, where the target underwent slight rotation, $MF_{P}$ failed to track the target, while $MF_{BASE}$ successfully maintained the target’s position, though with a less accurate bounding box. In the *ball2* sequence, $MF_{BASE}$ successfully tracked the target, whereas $MF_{P}$ failed. These examples illustrate specific cases where the performance of $MF_{P}$ and $MF_{BASE}$ diverge. It is worth noting, however, that in the majority of the cases, the results of $MF_{P}$ and $MF_{BASE}$ were nearly identical.

### 4.4. Discussion

The proposed method introduces variability in computation times due to the confidence evaluation mechanism, which dynamically determines whether lightweight tracking or a robust algorithm is applied for each frame. While this adaptive approach improves tracking performance, the variability in processing times can pose challenges in real-time control systems where consistent cycle times are critical. To address this limitation, the following potential solutions can be explored: (a) parameter optimization and (b) buffering or pipelining. In parameter optimization, adjusting the confidence threshold and fine-tuning the frequency of robust algorithm invocation could reduce processing time variability while maintaining performance. Implementing a buffering or pipelining strategy could smooth out fluctuations in computation time, ensuring more stable processing cycles in real-time systems. On the other hand, this variability offers a practical advantage in mobile and battery-powered applications. By primarily relying on lightweight tracking and invoking the robust algorithm only when necessary, the proposed method significantly reduces computational overhead and power consumption. This trade-off makes the method particularly suitable for devices such as drones, smartphones, and other mobile platforms where energy efficiency is critical. Further research could focus on optimizing the proposed method to reduce computation time variability while preserving its power-saving benefits, enabling broader applicability in real-time and resource-constrained environments.

The lightweight object tracking method employs the block-matching algorithm, and its tracking accuracy may degrade in challenging scenarios, such as severe occlusion, rapid object appearance changes, object disappearance, or highly cluttered backgrounds. In these situations, the confidence level typically drops below the predefined threshold, invoking the complex but robust tracker to ensure reliable tracking. However, this process increases the overall processing time, particularly in sequences where the robust tracker is frequently activated. For example, although the proposed method improved processing time in most test sequences, it slightly decreased the processing time for the *book* sequence, as shown in Table 4.

## 5. Conclusions

This study presented a method to accelerate object tracking speed by proposing a novel approach that combines a lightweight tracking algorithm with existing robust yet complex algorithms. Our approach intelligently applies the robust algorithm only when necessary. Thus, the proposed method significantly improves the tracking speed with a minor degradation in tracking accuracy. The proposed confidence level evaluation plays a crucial role in determining the tracking strategy for each frame, essentially ensuring that the robust algorithm intervenes only when the lightweight tracking method is unsuccessful. This innovation strikes a balance between computational efficiency and tracking quality. Our experiments validated the effectiveness of our approach by showing remarkable improvements in tracking speed while preserving accuracy. Moreover, the proposed approach’s flexibility in integration with existing methods demonstrates its potential for practical implementation. This study further presented integration examples highlighting the adaptability of this approach across various tracking scenarios. The proposed methodology not only contributes to the advancement of real-time tracking capabilities but also paves the way for more efficient utilization of computing resources in complex tracking environments. Further research could focus on optimizing the confidence level evaluation parameters and exploring additional ways to enhance the synergy between lightweight and robust tracking methods.

## Figures and Tables

**Table 1:** Performance of SiamRPN++ and LTS. EOA and LTS stand for expected average overlap and lightweight tracking with SiamRPN++.

Method	Accuracy	Robustness	Lost Count	EOA	SKIP (%)
SiamRPN++	0.604	0.243	52	0.413	0
LTS	0.598	0.445	95	0.277	48.7

**Table 2:** Comparisons of $M_{\mathrm{BASE}}$ (SiamRPN++ using ResNet backbone), frame skipping ($M_{\mathrm{SKIP}}$), and proposed method without the confidence level evaluation ($M_{P1}$) and with the confidence level evaluation ($M_{P2}$).

Method	$S_{N}$	Accuracy	Robustness	Lost Count	EOA	S(%)	FPS
$M_{\mathrm{BASE}}$	-	0.604	0.243	52	0.413	0	58.3
$M_{\mathrm{SKIP}}$	1	0.580	0.524	112	0.228	49.8	118.4
	2	0.564	1.025	219	0.124	66.3	170.8
	3	0.552	1.353	289	0.087	74.5	215.2
	4	0.540	1.573	336	0.082	79.4	253.3
	5	0.523	1.700	363	0.072	82.6	288.9
	10	0.485	2.154	460	0.056	90.0	428.2
$M_{P1}$	1	0.595	0.407	87	0.298	49.8	114.8
	2	0.582	0.632	135	0.194	66.4	161.0
	3	0.585	0.791	169	0.175	74.6	199.7
	4	0.579	0.791	169	0.173	79.6	232.7
	5	0.571	0.819	175	0.160	82.9	264.3
	10	0.549	1.054	225	0.133	90.4	392.0
$M_{P2}$ (T = 0.5)	1	0.603	0.276	59	0.389	34.1	88.2
	2	0.597	0.290	62	0.358	45.1	101.6
	3	0.595	0.304	65	0.356	50.2	111.2
	4	0.595	0.328	70	0.353	53.7	118.3
	5	0.588	0.332	71	0.341	55.6	123.9
	10	0.578	0.393	84	0.304	59.8	135.8

**Table 3:** Performance variation with respect to different threshold values. $S_{N}$ for $M_{P1}$ was set to 1.

Method	Thres.	Accuracy	Robustness	Lost Count	EOA	S (%)	FPS
$M_{\mathrm{BASE}}$	-	0.604	0.243	52	0.413	0	58.3
$M_{P1}$	-	0.595	0.407	87	0.298	49.8	114.8
$M_{P2}$ ($S_{N}$ = 1)	1.00	0.609	0.272	58	0.376	14.0	68.0
	0.67	0.605	0.243	52	0.415	26.8	76.6
	0.50	0.603	0.276	59	0.389	34.1	88.2
	0.40	0.601	0.276	59	0.380	38.5	91.0
	0.33	0.598	0.262	56	0.393	41.6	95.8
	0.25	0.597	0.332	71	0.331	45.2	102.1
	0.20	0.596	0.314	67	0.355	47.2	105.9

**Table 4:** Comparison of $M_{P2}$ with $M_{\mathrm{BASE}}$ in terms of FPS for each sequence. $S_{N}$ and T were 1 and 0.5, respectively.

Seq.	$M_{\mathrm{BASE}}$	$M_{P2}$	Seq.	$M_{\mathrm{BASE}}$	$M_{P2}$
ants1	51.9	59.7	graduate	57.1	86.5
ants3	63.7	76.8	gymnastics1	58	100.8
ball2	61	104.8	hand	59.3	85.7
basketball	63.3	88	handball1	59.7	117
birds1	61.6	60.1	handball2	59.2	101.3
bolt1	60	104.7	iceskater2	56.1	80.3
book	60.7	58.9	matrix	56.5	67.4
butterfly	55.9	62.3	motocross1	58.1	81.2
conduction1	59.7	109.2	nature	55.7	84.2
drone1	60.4	115.5	road	58.1	112.5
drone across	57.4	89.3	shaking	57.2	81.8
drone flip	61.9	98.1	sheep	57.8	113.6
fernando	57.2	69.9	singer2	54.5	82
fish1	59.1	115.3	singer3	55.5	68
fish3	58	114.2	soccer2	60.6	70.4
flamingo1	59.1	104.1	soldier	58.2	76.7
girl	58.8	109.9	traffic	58.4	114.8
glove	60.7	84.8	wiper	58.4	96.8

**Table 5:** Variation in performance across different threshold values. Here, $M_{\mathrm{Mobile}}$ denotes SiamRPN++ utilizing the MobileNet backbone. In $M_{P3}$, the lightweight object tracking method with confidence level evaluation is integrated with $M_{\mathrm{Mobile}}$.

Method	Thres.	Accuracy	Robustness	Lost Count	EOA	S(%)	FPS
$M_{\mathrm{BASE}}$	-	0.604	0.243	52	0.413	0	58.3
$M_{\mathrm{Mobile}}$	-	0.587	0.234	50	0.411	0	85.5
$M_{P3}$ ($S_{N}$ = 1)	1.0	0.580	0.304	65	0.349	14.0	95.7
	0.67	0.585	0.267	57	0.371	26.8	109.6
	0.50	0.586	0.290	62	0.352	34.2	122.0
	0.40	0.574	0.253	54	0.370	38.5	129.4
	0.33	0.587	0.267	57	0.384	41.5	140.4
	0.25	0.584	0.295	63	0.354	45.2	146.0
	0.20	0.583	0.309	66	0.339	47.1	151.0

**Table 6:** Comparisons of $M_{P2}$ (the proposed method with SiamRPN++), $MF_{P}$ (the proposed method with MixFormer [22]), and $U_{P}$ (the proposed with the ground truth) in terms of the skipping rate (%). The threshold was set to 0.5.

	Skipping Rate, S (%)								
	$S_{N}$ = 1			$S_{N}$ = 2			$S_{N}$ = 5		
**Seq.**	$M_{P2}$	${\mathrm{MF}}_{P}$	$U_{P}$	$M_{P2}$	${\mathrm{MF}}_{P}$	$U_{P}$	$M_{P2}$	${\mathrm{MF}}_{P}$	$U_{P}$
average	**0.34**	**0.34**	**0.34**	**0.44**	**0.45**	**0.45**	**0.55**	**0.55**	**0.56**
bag	0.39	0.40	0.40	0.51	0.52	0.53	0.63	0.65	0.65
basketball	0.35	0.33	0.38	0.48	0.45	0.51	0.59	0.56	0.65
bolt1	0.44	0.37	0.41	0.58	0.49	0.55	0.70	0.59	0.68
book	0.03	0.04	0.02	0.05	0.06	0.03	0.05	0.06	0.03
conduction1	0.42	0.34	0.46	0.53	0.43	0.60	0.71	0.54	0.76
fish1	0.49	0.49	0.49	0.63	0.65	0.65	0.78	0.81	0.82
girl	0.47	0.47	0.47	0.63	0.64	0.64	0.78	0.78	0.79
helicopter	0.49	0.49	0.49	0.64	0.66	0.66	0.78	0.82	0.82
leaves	0.02	0.00	0.02	0.02	0.00	0.03	0.02	0.00	0.08
singer2	0.39	0.39	0.37	0.51	0.52	0.49	0.64	0.67	0.61
tiger	0.12	0.15	0.11	0.13	0.18	0.15	0.16	0.22	0.17
traffic	0.50	0.49	0.50	0.66	0.66	0.66	0.83	0.83	0.83

**Table 7:** Comparisons of $M_{P2}$ (the proposed method with SiamRPN++), $MF_{P}$ (the proposed method with MixFormer), and $U_{P}$ (the proposed with the ground truth) in terms of accuracy, robustness, and EOA. The threshold was set to 0.5.

Method	$S_{N}$	Accuracy	Robustness	EOA
$M_{BASE}$	0	0.604	0.243	0.413
	1	0.603	0.276	0.389
$M_{P2}$	2	0.597	0.29	0.358
	5	0.588	0.332	0.341
$MF_{BASE}$	0	0.545	0.229	0.341
	1	0.577	0.215	0.374
$MF_{P}$	2	0.572	0.215	0.381
	5	0.589	0.281	0.340
$U_{BASE}$	0	0.788	0	0.795
	1	0.775	0	0.780
$U_{P}$	2	0.767	0	0.771
	5	0.736	0	0.737

**Table 8:** Comparisons of $MF_{BASE}$ and $MF_{P}$ (the proposed method with MixFormer) on OTB100 and UAV123 datasets. The threshold was set to 0.5. **P** stands for precision.

Method	$S_{N}$	OTB100			UAV123		
		**AUC (%)**	**P (%)**	**FPS**	**AUC (%)**	**P (%)**	**FPS**
$MF_{BASE}$	0	71.61	94.21	48.04	67.27	89.73	49.55
$MF_{P}$	1	70.93	92.70	80.12	67.89	89.89	92.50
	2	71.20	92.75	107.21	68.77	91.00	131.32
	5	69.31	90.70	174.79	67.92	90.10	207.91

## Data Availability

All data derived from this study are presented in the article.

## References

[1] Lee K.-H., Hwang J.-N. (2015). On-road pedestrian tracking across multiple driving recorders. IEEE Trans. Multimed..

[2] Lee K.-H., Hwang J.-N., Okopal G., Pitton J. (2016). Ground-movingplatform-based human tracking using visual slam and constrained multiple kernels. IEEE Trans. Intell. Transp. Syst..

[3] Lu X., Ma C., Ni B., Yang X. (2021). Adaptive region proposal with channel regularization for robust object tracking. IEEE Trans. Circuits Syst. Video Technol..

[4] Liu L., Xing J., Ai H., Ruan X. Hand posture recognition using finger geometric feature. Proceedings of the 21st International Conference on Pattern Recognition.

[5] Wu Y., Lim J., Yang M.-H. (2015). Object tracking benchmark. IEEE Trans. Pattern Anal. Mach. Intell..

[6] Dalal N., Triggs B. Histograms of oriented gradients for human detection. Proceedings of the IEEE Computer Society Conference on Computer Vision and Pattern Recognition (CVPR).

[7] Marvasti-Zadeh S.M., Cheng L., Ghanei-Yakhdan H., Kasaei S. (2022). Deep learning for visual tracking: A comprehensive survey. IEEE Trans. Intell. Transp. Syst..

[8] Han Z., Wang P., Ye Q. (2020). Adaptive discriminative deep correlation filter for visual object tracking. IEEE Trans. Circuits Syst. Video Technol..

[9] Chen K., Tao W. (2018). Once for all: A two-flow convolutional neural network for visual tracking. IEEE Trans. Circuits Syst. Video Technol..

[10] Li S., Zhao S., Cheng B., Zhao E., Chen J. (2020). Robust visual tracking via hierarchical particle filter and ensemble deep features. IEEE Trans. Circuits Syst. Video Technol..

[11] Nam H., Han B. Learning multi-domain convolutional neural networks for visual tracking. Proceedings of the IEEE Conference on Computer Vision and Pattern Recognition (CVPR).

[12] Zhu Z., Huang G., Zou W., Du D., Huang C. Uct: Learning unified convolutional networks for real-time visual tracking. Proceedings of the IEEE International Conference on Computer Vision Workshops.

[13] Han B., Sim J., Adam H. Branchout: Regularization for online ensemble tracking with convolutional neural networks. Proceedings of the IEEE Conference on Computer Vision and Pattern Recognition.

[14] Wang M., Liu Y., Huang Z. Large margin object tracking with circulant feature maps. Proceedings of the IEEE Conference on Computer Vision and Pattern Recognition.

[15] Pu S., Song Y., Ma C., Zhang H., Yang M.-H. (2018). Deep attentive tracking via reciprocative learning. Adv. Neural Inf. Process. Syst..

[16] Guo Q., Feng W., Zhou C., Huang R., Wan L., Wang S. Learning dynamic siamese network for visual object tracking. In Proceedings of the IEEE International Conference on Computer Vision.

[17] Li B., Yan J., Wu W., Zhu Z., Hu X. High performance visual tracking with siamese region proposal network. Proceedings of the IEEE Conference on Computer Vision and Pattern Recognition.

[18] Zhu Z., Wang Q., Li B., Wu W., Yan J., Hu W. Distractor-aware siamese networks for visual object tracking. Proceedings of the European Conference on Computer Vision (ECCV).

[19] Shan Y., Zhou X., Liu S., Zhang Y., Huang K. (2020). Siamfpn: A deep learning method for accurate and real-time maritime ship tracking. IEEE Trans. Circuits Syst. Video Technol..

[20] Yang T., Chan A.B. Recurrent filter learning for visual tracking. Proceedings of the IEEE International Conference on Computer Vision Workshops.

[21] Zhao F., Wang J., Wu Y., Tang M. (2019). Adversarial deep tracking. IEEE Trans. Circuits Syst. Video Technol..

[22] Cui Y., Jiang C., Wu G. (2024). Mixformer: End-to-end tracking with iterative mixed attention. IEEE Trans. Pattern Anal. Mach. Intell..

[23] Cui Y., Jiang C., Wang L., Wu G. Mixformer: End-to-end tracking with iterative mixed attention. Proceedings of the IEEE/CVF Conference on Computer Vision and Pattern Recognition.

[24] Li H., Wang X., Shen F., Li Y., Porikli F., Wang M. (2019). Real-time deep tracking via corrective domain adaptation. IEEE Trans. Circuits Syst. Video Technol..

[25] Hong S., You T., Kwak S., Han B. Online tracking by learning discriminative saliency map with convolutional neural network. Proceedings of the International Conference on Machine Learning. PMLR.

[26] Wang Q., Teng Z., Xing J., Gao J., Hu W., Maybank S. Learning attentions: Residual attentional siamese network for high performance online visual tracking. Proceedings of the IEEE Conference on Computer Vision and Pattern Recognition.

[27] Zhang P., Zhuo T., Huang W., Chen K., Kankanhalli M. (2017). Online object tracking based on cnn with spatial-temporal saliency guided sampling. Neurocomputing.

[28] Cheng K.W., Chan S.C. Fast block matching algorithms for motion estimation, In Proceedings of the 1996 IEEE International Conference on Acoustics, Speech, and Signal Processing Conference Proceedings, Atlanta, GA, USA, 9 May 1996.

[29] El-Azim S.A., Ismail I., El-Latiff H.A. An efficient object tracking technique using block-matching algorithm. Proceedings of the Nineteenth National Radio Science Conference.

[30] Gyaourova A., Kamath C., Cheung S. (2003). Block Matching for Object Tracking.

[31] Hariharakrishnan K., Schonfeld D. (2005). Fast object tracking using adaptive block matching. IEEE Trans. Multimed..

[32] Krizhevsky A., Sutskever I., Hinton G.E. (2012). Imagenet classification with deep convolutional neural networks. Adv. Neural Inf. Process. Syst..

[33] Simonyan K., Zisserman A. (2014). Very deep convolutional networks for large-scale image recognition. arXiv.

[34] Li B., Wu W., Wang Q., Zhang F., Xing J., Yan J. Siamrpn++: Evolution of siamese visual tracking with very deep networks. Proceedings of the IEEE/CVF Conference on Computer Vision and Pattern Recognition.

[35] Fu C., Lu K., Zheng G., Ye J., Cao Z., Li B., Lu G. (2023). Siamese object tracking for unmanned aerial vehicle: A review and comprehensive analysis. Artif. Intell. Rev..

[36] Ondrasovic M., Tarabek P. (2021). Siamese visual object tracking: A survey. IEEE Access.

[37] Ren S., He K., Girshick R., Sun J. (2015). Faster r-cnn: Towards real-time object detection with region proposal networks. arXiv.

[38] He K., Zhang X., Ren S., Sun J. Deep residual learning for image recognition. Proceedings of the IEEE Conference on Computer Vision and Pattern Recognition.

[39] Kristan M., Leonardis A., Matas J., Felsberg M., Pflugfelder R., Zajc L.C., Vojir T., Bhat G., Lukezic A., Eldesokey A. The sixth visual object tracking vot2018 challenge results. Proceedings of the European Conference on Computer Vision (ECCV) Workshops.

[40] MixFormer. https://github.com/MCG-NJU/MixFormer.

[41] PySOT. https://github.com/STVIR/pysot.

[42] Howard A.G., Zhu M., Chen B., Kalenichenko D., Wang W., Weyand T., Andreetto M., Adam H. (2017). Mobilenets: Efficient convolutional neural networks for mobile vision applications. arXiv.

[43] Mueller M., Smith N., Ghanem B. A benchmark and simulator for UAV tracking. Proceedings of the European Conference on Computer Vision.

